# Breast cancer metastasis to the stomach mimicking early gastric cancer

**DOI:** 10.1002/jgh3.12959

**Published:** 2023-08-26

**Authors:** Kosuke Tanaka, Yohei Yabuuchi, Daisuke Yamashita, Tetsuro Inokuma

**Affiliations:** ^1^ Department of Gastroenterology Kobe City Medical Center General Hospital Kobe Japan; ^2^ Department of Pathology Kobe City Medical Center General Hospital Kobe Japan

**Keywords:** breast cancer, early gastric cancer, metastasis

## Abstract

A 79‐year‐old woman with a history of invasive lobular breast cancer presented with a lesion that was endoscopically and histopathologically consistent with poorly differentiated early gastric adenocarcinoma. Endoscopic submucosal dissection was performed, and histopathological examination using additional immunohistochemistry determined that the lesion was metastatic breast cancer. Even if a lesion suspicious of gastric cancer is found on endoscopy in a patient with a history of breast cancer, the possibility of metastasis should be considered and clinicians should inform the pathologists of the possibility.

## Graphical abstract

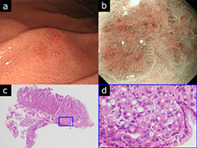

## Introduction

The most common sites of breast cancer metastasis are the skeleton, lungs, and liver, and the incidence of metastasis to the stomach has been estimated at 2%–18%.^1^ Gastric metastases of breast cancer have varying endoscopic manifestations and may resemble a submucosal tumor, mucosal erosion, early gastric cancer, or advanced gastric cancer (especially the linitis plastica type).^1^, ^2^, ^3^ Distinguishing between gastric metastasis of breast cancer and primary gastric cancer is clinically important because these lesions require different treatment strategies. Here we report a case of breast cancer metastasis to the stomach mimicking early gastric cancer.

## Case report

**Figure 1:**
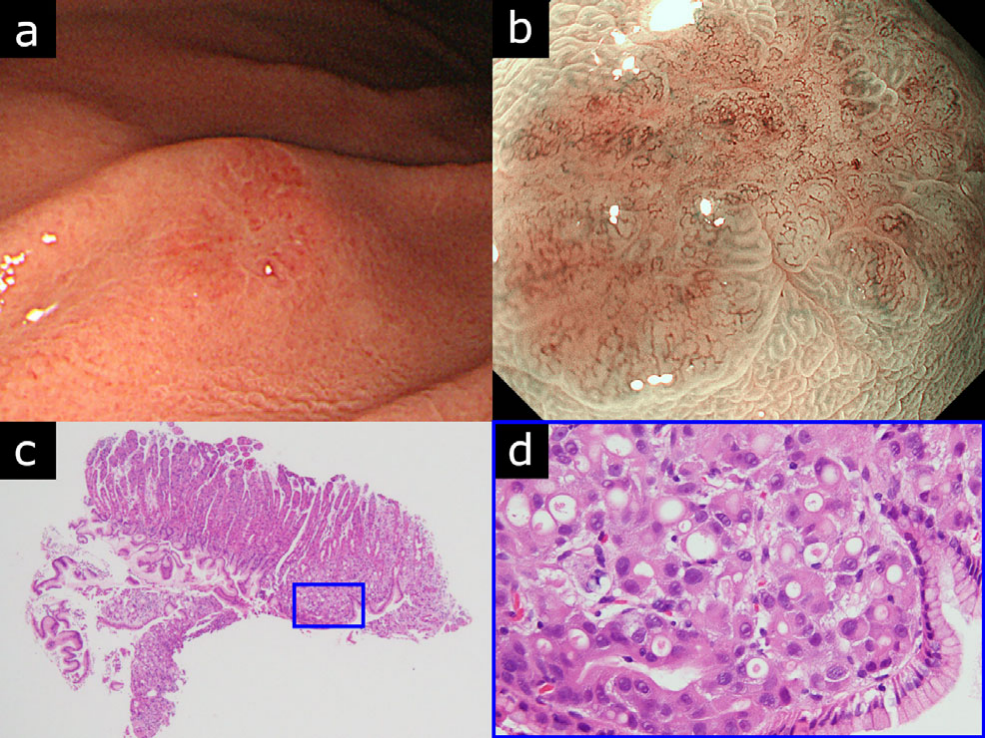
(a) A 7‐mm reddish depressed lesion in the greater curvature of the upper gastric body of the stomach. (b) Magnifying endoscopy with narrow‐band imaging revealed tortuous dilated non‐loop microvessels with a demarcation line. (c, d) Biopsy specimen on histopathology confirmed poorly differentiated adenocarcinoma and signet ring cell carcinoma (hematoxylin–eosin stain).

A 79‐year‐old woman presented with epigastric discomfort during a follow‐up. She had previously undergone partial mastectomy for left invasive lobular breast cancer (T2N0M0 Stage IIA, according to the eighth edition of the UICC for the International Cancer Control staging system), followed by a 5‐year adjuvant hormonal therapy with toremifene. Subsequently, she underwent five surgeries for local recurrences (one in the axillary lymph node, two in the preserved breast, and two subcutaneous recurrences in the left chest). Esophagogastroduodenoscopy revealed a 7‐mm reddish depressed solitary lesion in the greater curvature of the upper gastric body of the stomach (Fig. 1a). Magnifying endoscopy with narrow‐band imaging (NBI) revealed tortuous dilated non‐loop microvessels with a demarcation line (Fig. 1b). Biopsy specimen confirmed poorly differentiated adenocarcinoma and signet ring cell carcinoma (Fig. 1c,d). We diagnosed this lesion as early gastric cancer indicated for endoscopic resection. Contrast‐enhanced computed tomography was performed before endoscopic resection, revealing no obvious metastatic lesions. Endoscopic submucosal dissection (ESD) was performed and histopathological examination also revealed poorly differentiated adenocarcinoma and signet ring cell carcinoma infiltrating the submucosa with lymphovascular invasion (Supplementary Fig. 1a–c). Considering her history of breast cancer, it was necessary to rule out metastatic breast cancer. Additional examination using immunohistochemistry showed that the tumor cells were positive for GATA binding protein 3 and Gross Cystic Disease Fluid Protein 15 (Supplementary Fig. 1d,e), which are diagnostic markers for mammary differentiation in histopathology.^4^, ^5^ The test results for estrogen receptor, progesterone receptor, and cytokeratin 20 were negative. Finally, this case was diagnosed as metastatic breast cancer. Within a month after the ESD, computed tomography revealed axillary lymphadenopathy, suggesting metastatic breast cancer. The patient received chemotherapy but died 20 months later.

## Discussion

Our endoscopic diagnosis of early gastric cancer was based on a magnifying endoscopy simple diagnostic algorithm for early gastric cancer (MESDA‐G).^6^ Early gastric cancer can be diagnosed by the presence of a demarcation line and an irregular microvascular or microsurface pattern.^6^ Additionally, we predicted the histological subtype based on the following criteria.^7^, ^8^ In magnified NBI images, a fine network pattern and intralobular loop pattern are often observed in differentiated early gastric cancer. In contrast, a corkscrew pattern is often observed in poorly differentiated early gastric cancer. In this case, we suspected that the lesion was a poorly differentiated early gastric cancer.

In this case, both endoscopic and histopathological findings of the lesion closely resembled poorly differentiated early gastric adenocarcinoma. Since it is difficult to distinguish between primary gastric cancer and gastric metastasis of breast cancer using hematoxylin–eosin staining alone, detailed immunohistochemistry is essential for diagnosis.^1^ If immunohistochemistry had not been performed on the ESD specimen, the diagnosis would have been primary gastric cancer. As this lesion fulfilled the eCure C‐2 criteria according to the Japanese Gastric Cancer Association guidelines,^9^ additional surgical resection would have been recommended, potentially causing unnecessary invasion. As the patient's history of breast cancer was shared with the pathologist, additional immunostaining led to the diagnosis of breast cancer, avoiding unnecessary surgery. Therefore, even if a lesion suspicious of gastric cancer is found on endoscopy in a patient with a history of breast cancer, the possibility of metastasis should be considered and clinicians should inform the pathologists of the possibility.

### Informed consent statement

Informed consent was obtained from the patient to publish these images.

## Supporting information


**Supplementary Figure 1.** (a, b, c) Poorly differentiated and signet ring cell carcinoma infiltrated the submucosa with lymphovascular invasion (hematoxylin–eosin stain). (d) The tumor cells were positive for GATA binding protein 3. (e) The tumor cells were positive for Gross Cystic Disease Fluid Protein 15.
