# Supplementary figures and images for: Breast cancer metastasis to the stomach mimicking early gastric cancer
Source: JGH Open. 2023 Aug 26;7(9):667–8. doi: 10.1002/jgh3.12959 (PMC10517435; doi:10.1002/jgh3.12959)

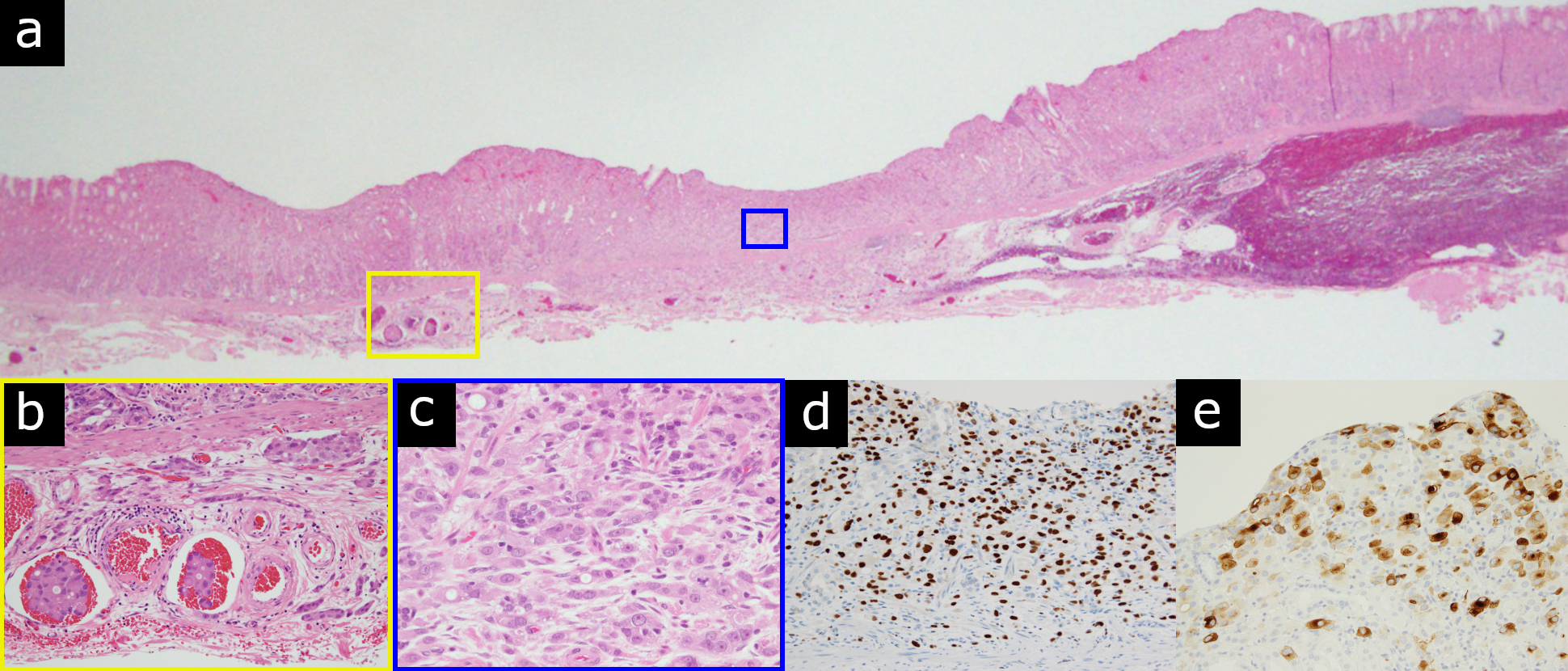

Supplement: Supplementary file 1 — Supplementary Figure 1. (a, b, c) Poorly differentiated and signet ring cell carcinoma infiltrated the submucosa with lymphovascular invasion (hematoxylin–eosin stain). (d) The tumor cells were positive for GATA binding protein 3. (e) The tumor cells were positive for Gross Cystic Disease Fluid Protein 15. [file JGH3-7-667-s001.tif]
